# On the ambiguity regarding the relationship between sequential congruency effects, bilingual advantages in cognitive control, and the disengagement of attention

**DOI:** 10.3934/Neuroscience.2019.4.282

**Published:** 2019-11-01

**Authors:** Kenneth R. Paap, Hunter Myuz, Regina Anders-Jefferson, Lauren Mason, Brandon Zimiga

**Affiliations:** 1 Language, Attention, and Cognitive Engineering Laboratory, Department of Psychology, San Francisco State University, San Francisco, CA, United States; 2 Department of Psychology, New Mexico State University, Las Cruces, NM, United States

**Keywords:** bilingualism, executive attention, disengagement of attention, sequential congruency effects, cognitive control

## Abstract

Grundy, Bialystok, and colleagues have reported that at short response-stimulus intervals bilinguals have smaller sequential congruency effects in flanker tasks compared to monolinguals. They interpret these differences to mean that bilinguals are more efficient at disengaging attentional control. Ten empirical studies are presented that show no differences between bilinguals and monolinguals under conditions that produced robust sequential congruency effects. These null results are discussed with respect to the rate at which sequential congruency effects dissipate and the fact these effects are not adaptive in the sense of improving overall performance. Arguments made by Goldsmith and Morton [1] that smaller sequential congruency effects should not be interpreted as “advantages” are extended. Evidence is also presented that neither simple congruency effects, nor sequential congruency effects, correlate across tasks. This lack of convergent validity is inconsistent with the hypothesis that either provides a measure of domain-general control that could underlie an advantage accrued through experience in switching languages. Results from other tasks purporting to show bilingual advantages in the disengagement of attention are also reviewed. We conclude that sequential congruency effects in nonverbal interference tasks and differences in the rate of disengaging attention are unlikely to contribute to our understanding of bilingual language control and that future research might productively examine differences in proactive rather than reactive control.

## 1. Introduction

The controversy regarding the hypothesis that bilinguals have better executive functioning (EF) has shifted from the EF components of inhibition, switching, and updating to hypothesized advantages in executive attention [2]. The purpose of this review is to focus on a recent set of empirical articles [3],[4] and ensuing critiques [1],[5]–[8] regarding the proposition that the magnitude of sequential congruency effects (SCEs) in nonverbal interference tasks such as the flanker task can be used as a valid and reliable measure of the ability to disengage attention and reveals an important consequence of bilingual experience. The controversy begins with Grundy et al.'s [3] finding that bilinguals had smaller SCEs than monolinguals in two of their experiments.

**Figure 1.**
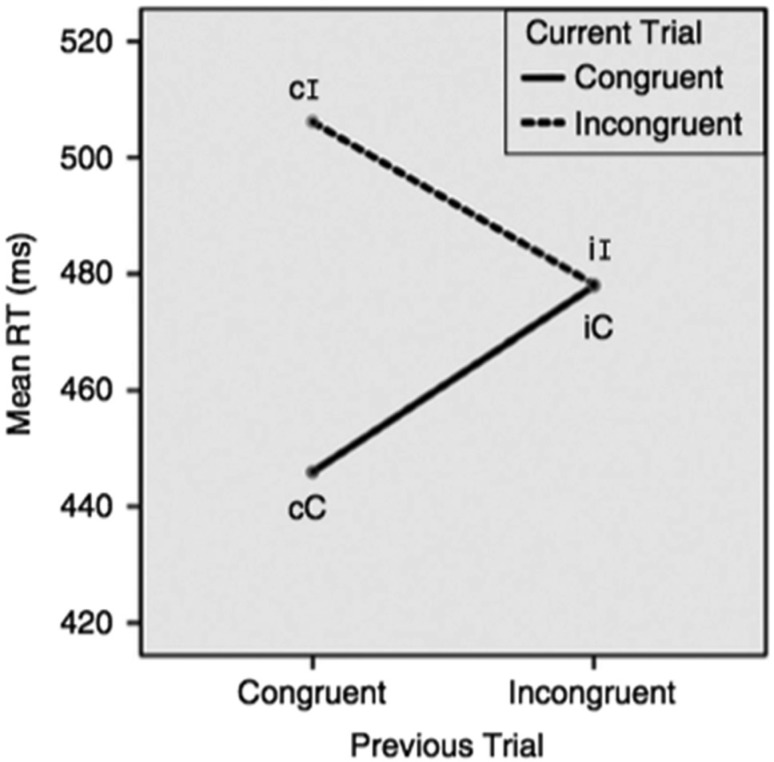
The SCE derived from the Simon task in Paap and Greenberg [9].

SCEs are trial-to-trial sequence effects that occur in most nonverbal interference tasks and are defined as smaller interference effects when the previous trial is incongruent compared to when it is congruent as shown in Figure 1 and operationally defined as: SCE = (cI – cC) – (iI – iC), where lowercase letters represent the previous trial and uppercase letters the current trial.

The groundwork for this debate is laid by Grundy, et al. [3] when they hypothesized *“that bilinguals can more rapidly disengage attention from irrelevant information than monolinguals in a simple flanker task”* (p. 42). On an incongruent trial of a simple flanker task, the central target arrow might point to the right while the irrelevant flankers point to the left. Ceteris paribus, individuals who can quickly focus attention on the center location will respond faster on these incongruent trials and have smaller interference scores than those who focus attention more slowly or broadly. But, as Grundy et al. [3] concede, and many recent meta-analyses confirm [5],[10],[11] bilinguals do not have smaller interference scores compared to monolinguals.

## 2. The Grundy et al. account of sequential congruency effects

The hypothesis that bilinguals can more rapidly disengage attention from irrelevant information (e.g., the irrelevant flankers in the flanker task) was recast to whether they can more rapidly disengage from the cognitive control required on the previous trial.

There are many explanations for SCEs (see [12] for a discussion of the various accounts), and in the standard flanker task it is likely that multiple mechanisms are in play. However, a reviewer of an early version of this commentary, who has expertise in SCEs, observed that Grundy et al.'s [3] attentional engagement account is hard to align with any existing account of the SCE. The Grundy et al. account appeals to a shift in attention from the previous trial to the current trial, but does not describe a specific mechanism for generating sequential effects. Shifting from a stimulus in one physical location to another in a different location appeals to the traditional spotlight metaphor, but what seems to be required by the disengagement hypothesis (as applied to SCEs) is a shift from a stimulus array at one point in time to a new array that appears later. The following provides an example of what the disengagement hypothesis could be, but because we generated it, Grundy et al. may well prefer an alternative. One might assume that attention control is exercised within a single trial when the detection of conflict between the flankers and target triggers an upregulation of the relevant information and/or the down-regulation of the irrelevant information. Thus, the attentional control triggered and implemented on trial n-1 is not a preparation for trial n, but an unintended carryover effect from trial n-1. If the trial type happens to repeat, iI or cC, the carryover will benefit the decision on trial n, but it will produce a cost if the trial type switches (cI or iC). In this scenario disengagement would be a shift away from the control plan generated on the previous trial to a neutral state that could be rapidly adapted to the type of trial (congruent or incongruent) that occurs next. Rapid disengagers would be in a neutral state more often than slow disengagers and would therefore have smaller SCEs. As a reviewer, Grundy adds that disengagement is not necessarily an all or nothing phenomenon, but rather it may be a matter of degree.

The possibility that SCEs could be generated by the passive carry-over of control settings was discussed by Mayr and Awe [13]. Based on a model of task switching developed by Gilbert and Shallice [14], Mayr and Awe reported that typical SCEs can be simulated by passively carrying-over the control settings from one trial to the next because “surviving” a high-conflict trial resulted in the system automatically settling into a more controlled state than in “surviving” a low-conflict trial.

## 3. Language group differences reported by Costa, Hernández, & Sebastián-Gallés [15]

Arguably, Grundy and Bialystok were not the first to report differences between bilinguals and monolinguals in trial-to-trial sequence effects given Costa et al.'s [15] exploration of the cost of “switching” into congruent (or incongruent) trials in 2008. Costa et al. reported separate ANOVAs for current trials that are congruent and incongruent. Each ANOVA included Group (bilingual versus monolingual) and Previous Trial (no switch versus switch). The only significant Group x Previous Trial interaction was obtained on the analysis of current congruent trials, F(1, 198) = 4.03, *p* = .046 showing that the switching costs (i.e., iC trials minus cC trials) for bilinguals (about 9 ms) were smaller than the costs for monolinguals (about 17 ms). The interaction was not significant for the analysis of incongruent trials and showed switching costs of about 8 ms for both groups. The most diagnostic test for a group difference in the magnitude of CSEs, the three-way Group x Previous Trial x Current Trial interaction, was not conducted. It does appear, however, that Costa et al. [14] have captured a group difference on the “bottom half” of the CSE depicted in Figure 1 as bilinguals show smaller costs compared to monolinguals when a current congruent trial has been preceded by an incongruent trial rather than a congruent trial. If CSEs actually reflect conflict adaptation one might have expected the group differences to appear more prominently when participants actually have to resolve a conflict, that is, on current incongruent trials. But as reported above, the interaction was not significant in the analysis of incongruent trials.

## 4. Do bilinguals consistently show smaller SCEs compared to monolinguals?

Paap [5] briefly described SCEs derived from both flanker and Simon tasks across three laboratories and a total of ten experiments. Table 1 provides the descriptive statistics for these ten plus the more recent results from Goldsmith and Morton [4]. The magnitude of the SCEs was substantial in most of the 11 comparisons, but there were no significant group differences, and, in fact, most of the non-significant numerical trends actually showed smaller SCEs for the monolingual group (see Table 1). It is clear that when substantial SCEs are observed, significant group differences are rare and, in fact, restricted to the first two studies reported by Grundy et al. [3]. As the studies by Guido Mendes [16] (a dissertation) and Antón and Duñabeitia have not been published and passed the rigors of peer review it is worthwhile to describe their designs and results in further detail.

### 4.1. Data availability statement

The data used to compute SCEs from the Paap and Sawi (2014) study and the data used to compute the intertask correlations is available upon request from the corresponding author.

### 4.2. SCEs for bilinguals and monolinguals in Guido Mendes

Guido Mendes [16] tested for bilingual advantages (N = 115) across multiple measures derived from both a Simon task and the Attentional Network Test (ANT) that included the flanker effect as one of the test networks. All participants were immigrants to New Zealand and the groups were matched on SES, education, intelligence test scores and other factors. The bilinguals spoke a variety of L1s and were all proficient in English. The specific instantiation of the ANT was identical to the version used by Costa, Hernández, Costa-Faidella, and Sebastián-Gallés [17] to maximize conflict-monitoring demands. This version has no neutral trials and an equal proportion of congruent and incongruent trials. The version of the Simon task was identical to the one used by Bialystok et al., [18], Study 2 and includes conditions that either involve conflict (target presented to the side) or not (target centered) and also includes conditions that impose a low working memory load (a simple rule with a single target for each response) or a higher load (a rule that maps two targets to each response). Thus, the specific versions of each task logically and empirically supported bilingual advantages in earlier seminal studies.

**Table 1.** Studies showing no significant differences between bilinguals and monolinguals in the magnitude of SCEs in nonverbal interference tasks.

	Trial Type		c Effect	Trial Type		i Effect	**SCE**	Effect	Global
	cC	cI	cI-cC	iC	iI	iI-iC	c Ef.- I Ef.	I-C	RT
Paap & Greenberg (2013, [9])									
Simon									
Monolingual	450	503	53	480	477	−2	**55**	25	477
Bilingual	442	510	68	476	479	3	**65**	36	477
Flanker									
Monolingual	526	631	105	542	623	81	**24**	93	580
Bilingual	533	648	114	550	630	80	**34**	97	590
Paap & Sawi (2014, Session 1, [19])									
Simon									
Monolingual	435	491	56	464	458	−5	**61**	25	462
Bilingual	439	502	64	475	481	6	**58**	35	474
Flanker									
Monolingual	488	580	93	499	578	80	**13**	86	536
Bilingual	500	583	83	513	576	63	**20**	73	543
Paap & Sawi (2014, Session 2, [19])									
Simon									
Monolingual	423	475	52	447	453	6	**46**	29	462
Bilingual	423	481	57	452	462	10	**48**	34	474
Flanker									
Monolingual	474	554	80	476	549	73	**7**	77	513
Bilingual	469	540	72	474	539	66	**6**	69	505
Guido Mendes (2015, [16])									
Simon									
Monolingual	444	490	46	477	468	−9	**55**	19	470
Bilingual	440	491	51	478	471	−7	**58**	22	470
Flanker									
Monolingual	464	571	107	479	570	91	**16**	99	521
Bilingual	485	592	107	498	581	83	**24**	95	539
Antón & Duñabeitia (unpublished)									
Simon									
Monolingual	465	515	50	493	475	−18	**68**	16	487
Bilingual	406	480	74	466	445	−21	**95**	27	449
Flanker									
Monolingual	399	437	38	399	443	44	**-6**	41	420
Bilingual	384	433	49	387	423	36	**13**	43	407
Goldsmith & Morton (2018,[4])									
Flanker									
Monolingual	405	482	77	420	483	63	**14**	70	447
Bilingual	421	504	83	433	493	60	**23**	71	462

Note: c = previous trial was congruent, C = current trial was concurrent, i = previous trial was incongruent, I = current trial was incongruent, Ef. = effect.

The results were consistently null. In both tasks and across both speed and accuracy there were no differences between monolinguals and bilinguals on any of the measures analyzed: global RT, global accuracy, congruency effects, alerting effects, orienting effects, working memory costs, and SCEs. In the flanker task the magnitude of the SCE was +22 ms reflecting a reduction in the congruency effect from 107 ms when the previous trial was congruent to 85 ms when it was incongruent. In the Simon task the magnitude of the SCE was +58 ms reflecting a reduction in the congruency effect from 50 ms when the previous trial was congruent to −8 ms when it was incongruent. In both tasks the benefits on iI trials were approximately equal to the costs on iC trials. Most important for present purposes, the magnitude of the SCE were the same for both language groups.

### 4.3. SCEs for bilinguals and monolinguals in Antón and Duñabeitia

Ninety monolinguals and 90 bilinguals were tested in a variety of tasks including the Simon and Flanker. All the participants were from different regions of Spain (bilinguals from the Basque Country and monolinguals from Murcia) and were matched on age, sex, IQ and SES. Furthermore, all of them were non-immigrants and highly educated (88 out of 90 bilinguals and 87 out of 90 monolinguals possessed a university degree or were studying for one at the time they were tested). Bilinguals and monolinguals didn't differ in their Spanish mastery as measured by the LexTale task and all the bilinguals were native Basque speakers who acquired Spanish before the age of 6.

Both the Simon and flanker task (arrow version) were similar in procedure: participants were presented with a 1000 ms fixation point, after which the critical target was presented on the screen for 5000 ms or until a response was given. In the Simon task, participants saw either congruent, incongruent, or neutral (the item appeared in the center of the screen) trials. The flanker task also featured the same conditions: congruent, incongruent, or neutral (only the central arrow was displayed).

In both tasks, there were 16 congruent, incongruent, and neutral trials. In computing the four condition means for a CSE (cC, cI, iC, and iI) trials following a neutral trial were eliminated, as was the first trial, trials following errors, and outliers. If this led to zero valid observations in any condition that participant was dropped from the analysis. This resulted in 83 bilinguals and 85 monolinguals for the flanker analysis and 86 bilinguals and 87 monolinguals for the Simon analysis. Large CSEs were observed in the Simon task for both bilinguals (94.0) and monolinguals (67.2), but the group differences were non-significant, t(172) = 1.08, *p* = .86. The CSEs in the flanker task were very small for both bilinguals (13.7) and monolinguals (−5.6) and did not differ from each other, t(167) = 0.88, *p* = .19.

### 4.4. Identification of bilinguals and monolinguals in Paap's work

Some critics of our earlier work on the bilingual advantage hypothesis have raised concerns that we have relied on self ratings of language proficiency to form our language groups. It is certainly fair to point out that these two studies did not include objective measures of language proficiency, but we had far more background information to inform our partitioning than is generally acknowledged. Participants rated their proficiency in each language they were exposed to on a scale of 0 to 7 where 6 indicated as fluent as a typical native speaker and 7 better than a typical native speaker. They used this scale to rate their proficiency in speaking, listening, writing, and reading. As many of the participants raised in San Francisco's Asian-American communities have minimal reading and writing skills but enjoy high levels of fluency in speaking and listening we use a first-pass cut-off of 4.0 (based on an average of self-rated speaking and listening) to qualify as ‘bilingual’. If a participant had a lower mean, say 3.5, but indicated that they use a non-English language every day and that they often switch languages we considered background information to be ambiguous and deleted their data from the analyses based on groups. We also routinely conducted and reported supplementary analyses based on “extreme groups”, namely monolinguals who self-rated their exposure to a second language as a 0 or 1 and bilinguals who rated their L2 proficiency as either a 6 (as good as a native speaker) or a 7 (better than a native speaker). The null results we typically observe between language groups were never overturned in the extreme-group analyses and, in fact, the means in the two types of analyses tend to closely align.

As a further check we routinely analyze our data using regression so that L2 proficiency and the L2/L1 ratio can be treated as a continuous variable. These regression analyses also included as predictors other aspects of bilingual experience: age-of-acquisition of L2, percentage of English use, frequency-of-switching per day, frequency of switching within utterances, number of languages spoken per context (e.g., at home, at the university, at work, with friends, etc.). These regression analyses also provided no evidence that specific types of bilingual experience are associated with advantages in performance across a wide variety of measures of EF.

Self-ratings are highly correlated with a range of objective and standardized measures of language proficiency. For example, a study by Marian, Blumenfeld, and Kaushanskaya [20] correlated self-report measures of reading, speaking, and listening proficiency with eight different standardized measures of language skill involving reading, writing, speaking, and listening and covering both comprehension and production. These correlations were obtained for both L1 and L2. For L2 (the proficiency of greatest concern in classifying an individual as bilingual), all 24 correlations between the three subjective measures and the eight objective measures were significant with Pearson r values ranging from 0.29 to 0.74 with a mean of 0.59. Taking all of their results into account Marian et al. concluded that self-ratings are *“an effective, efficient, valid, and reliable tool for assessing bilingual language status.”* (p. 960). Likewise, Francis and Strobach [21] reported that self-ratings in both English and Spanish are highly predictive of standardized objective measures.

In other studies conducted in our lab using the same population of student participants and the same recruiting methods self-rated English proficiency significantly predicted performance in: (a) the multilingual naming task, (b) a sentence comprehension task requiring resolution of lexical ambiguity (c) judging if sentences contain a semantic anomaly, (d) judging if sentences contain a syntactic error, (e) deciding if letter strings are English words or non-words, (f) category fluency (number of correct responses to a category probe), and (g) reading time to a critical word in sentences with a semantic anomaly or syntactic error. Finally, to contrast our studies with those of Grundy et al. [3] although they did have participants take a test of English receptive vocabulary they had no objective measure of L2 proficiency and apparently relied on self ratings to identify their monolinguals and bilinguals.

## 5. The Time-Course of sequential congruency effects

In addition to the 12 earlier experiments listed in Paap [5], Goldsmith and Morton [4] also reported a failure to replicate the finding that bilinguals have smaller SCEs compared to monolinguals. The main counterargument pressed by the Grundy and Bialystok [8] commentary on Goldsmith and Morton [4] is that the failure to replicate was no-such-thing because Goldsmith and Morton only used response-stimulus intervals (RSIs) of about 1000 ms, and this is in the range where Grundy et al. [3] showed unreliable differences in SCEs between bilinguals and monolinguals. The general point is valid, Goldsmith & Morton's [4] failure to reproduce group differences would have greater impact if the replication had used the shorter RSIs where the differences in Grundy et al. [3] were more robust. However, this general point about the value of exact replication becomes moot because the underlying logic of the hypothesized bilingual “advantage” in SCEs appears to suffer from a logical fallacy when it confronts the full pattern of data.

Consider the assumption that as RSI increases the effects of attention control dissipate. In discussing the absence of group differences in RT in their Experiment 3, Grundy et al. [3] assert that *“the experiment required long RSIs during which all participants would have had sufficient time to disengage attention”* (p. 51). Now, if SCEs are generally due to the effects of the control exercised on one trial carrying over to the next trial, but if the Experiment 3 RSIs are sufficiently long for “all participants” to disengage attention, then the SCEs should be near zero for both groups. In contrast to this expectation, Grundy et al. [3] reported a highly significant Previous-Trial Congruency by Current-Trial Congruency interaction, *F*(1, 109) = 42.10, *p* < 0.001 at the 1000 ms RSI across language groups. In other words, the SCE of 21 ms was robust. Likewise, the 20 ms SCE reported by Goldsmith and Morton [4] was highly significant, *F*(1, 63) = 14.6, *p* < .001. These results are simply inconsistent with the assertion that by 1000 ms of RSI “*all participants*” have disengaged from the previous trial.

If robust SCEs at RSIs of 1000 ms are taken as evidence that disengagement has yet to occur for most participants, then Grundy et al.'s model predicts a consistent bilingual advantage because more bilinguals should have disengaged in comparison to monolinguals. Thus, all the null results shown in Table 1 represent failed predictions. However, in his review of this article Grundy speculates that adding an assumption that disengagement occurs gradually rather than all-or-none can explain how group differences can disappear (e.g. as in their Experiment 3) while robust CSEs remain. The underlying logic of this argument is not intuitive. If (at some specific RSI) more bilinguals have disengaged attention in all-or-none fashion, or alternatively if bilinguals get further down the road of disengagement, the outcome should be the same. When there are robust CSEs, those for bilinguals should be smaller. When CSEs have entirely dissipated, there will be no group differences. The experiments reported in Table 1 all show robust CSEs and, as such, are inconsistent with Grundy et al.'s hypothesis that bilinguals disengage attention more quickly than monolinguals.

We have argued that the Grundy et al. [3] account of when they did—and when they did not—observe group differences in SCEs may be flawed because their longer RSI of about 1000 ms still produced robust SCEs. This does not mean that SCEs do not decay as the time between trials increases. The most systematic investigation of the temporal dynamics of SCEs were the two experiments reported by Egner, Ely, and Grinband [22] using a face-word Stroop task where participants made gender decisions about the face appearing on each trial. SCEs did decline as either ISI or RSI increased, but significant SCEs were obtained at 3,000 ms RSI in Experiment 1 and 2,000 ms ISI in Experiment 2. SCEs did eventually fall to zero, but not until delays in the range of 2.5 seconds (Experiment 2) to 6 seconds (Experiment 1). Furthermore, Egner et al. [22] also cite several neuroimaging studies that show robust SCEs when the RSI is jittered in the range of 3.0 to 6.0 seconds. In summary there is very little supporting evidence from other labs that bilinguals have smaller SCEs than monolinguals, or that robust SCEs dissipate at RSIs of about 1,000 ms.

## 6. Is the magnitude of the SCE related to overall task performance?

As established above, most of the time the magnitude of SCEs does not differ across language groups, but sometimes bilinguals have smaller SCEs. However, when bilinguals do have smaller SCEs (i.e., two of the experiments from Grundy et al. [3]) they are not accompanied by faster global RTs or smaller flanker effects. This should not come as a surprise because as Figure 1 shows the gain on iI trials is symmetrically cancelled by the cost on iC trials and, consequently, the overall RTs are the same regardless of whether the previous trial was congruent or incongruent. Assuming an equal number of congruent and incongruent trials, there is no change in global RT regardless of whether the magnitude of the SCEs is large, small, or even zero. This line of reasoning hurtles to the conclusion that controlling the magnitude of the SCE (through disengagement of attention or any other mechanism) is irrelevant to optimizing (or simply improving) overall performance.

Grundy et al. [3] suggest that the results of an experiment by Mayr and Awh [13] provides evidence for the hypothesis that smaller SCEs (presumably caused by more rapid disengagement of attention) indicate greater ‘efficiency’. Mayr and Awh reported that the significant SCEs observed in the first two blocks of a color-Stroop task disappear in blocks 3 through 10. Thus, with more extended practice performance improves and SCEs grow smaller and dissipate. Should this association be interpreted as an adaptive relationship between the magnitude of SCEs and overall performance such that smaller SCEs reflect enhanced efficiency (more rapid disengagement of attention) and faster global RT? Another aspect of the Mayr and Awh [13] study suggests that it should not. The participants were randomly assigned to three groups that received different proportions of congruent and incongruent trials. As expected the Stroop effect increased as the proportion of congruent trials increased and this effect was stable across the 10 blocks. That is, in blocks 3 to 10 robust block-wise effects were found in the absence of trial-to-trial sequence effects.

The full pattern of results reported by Mayr and Awh [13] fits well with Braver's [23] distinction between reactive and proactive control. Proactive control relies upon the anticipation and prevention of interference before it occurs, whereas reactive control relies upon the detection and resolution of conflict after its onset. In reactive control attention is recruited only as needed and in a just-in-time manner. It is the type of control that triggers trial-to-trial sequence effects. Proactive control is a form of “early selection” that maintains and sustains a plan to bias attention, perception, and action systems to favor goal relevant information and goal attainment. In Braver's dual mechanisms of control (DMC) framework the balance between the two types of control will be affected by the statistical regularities in the environment and individual differences. The Mayr and Awh [13] results can be interpreted as follows. Early on participants rely on a mix of proactive and reactive control and consequently will show significant SCEs and a proportion-congruent block-effect. Because proactive control is adaptive (e.g., it accepts the risk of greater interference when incongruent trials are relatively rare) all three groups settle into a strongly proactive mode tuned to the proportion they have been experiencing. Thus, the dominance of proactive control reduces the influence of reactive control and eliminates the SCEs. If this plausible interpretation of the Mayr and Awh experiment is a good one, then there is no reason to assume that the disappearance of SCEs is due to disengagement of attention in the Grundy et al. [3] sense.^1^

Bilinguals are not better at adjusting their attentional control in order to optimize their overall performance on the task. They do not have faster global RTs. They do not show smaller interference effects. They simply show smaller SCEs in two experiments that fail to replicate in more than a dozen others. Any interpretation of those language-group differences framed as a bilingual advantage in attentional control is specious. To be fair, Grundy et al. [3] never explicitly refer to smaller SCEs as bilingual advantages, but at the NSF sponsored workshop on bilingualism and executive function (at the CUNY Graduate Center) Grundy and Bialystok [25] presented a poster titled *“It's not that simple: Sequential congruency effects reveal a bilingual disengagement advantage”*. Although they later chose to eschew the term advantage they cite the Mayr and Awh experiments as showing that *“better disengagement results in more efficient performance over time”*. They also assert that the smaller SCEs advantages for bilinguals demonstrate that SCEs are more sensitive measures compared to overall flanker effects. Regardless of Grundy et al.'s [3] characterization, it seems worthwhile, in general terms, to consider if small magnitudes of SCEs should be considered good, bad, or indifferent. When significant SCEs are symmetrical and when they dissipate quickly with practice, indifferent appears to be the best option.

## 7. Sequential congruency effects are task specific

SCEs, like simple congruency effects, also appear to be task specific. They do not occur when two different nonverbal interference tasks are alternated [26] and this implies that the mechanism causing SCEs in one task are different from the mechanism in the other task. Furthermore, the magnitude of SCEs in the Simon task does not correlate with that obtained in the flanker task across three of our datasets [9],[26]. Whitehead, Brewer, and Blais [27] also showed that SCE magnitudes do not correlate when the same participants completed Simon, flanker, and Stroop tasks. This lack of convergent validity across tasks is inconsistent with any hypothesis that assumes a domain-general attention-control mechanism drives SCEs. In summary, neither simple congruency effects nor SCEs appear to be domain-general measures of cognitive control. Rather they appear to be task specific. Thus, there is no reason to expect that any of these nonverbal effects should be affected by the amount or type of bilingual experience.

## 8. Linking SCEs in nonverbal interference tasks to bilingual language control

Grundy et al.'s [3] intention to connect attentional disengagement in bilingual language control to domain-general attentional control begs this question: What logic or evidence suggests that disengagement of attention from the non-target language involves the same domain general mechanism involved in disengaging from the previous trial in the arrows-version of a flanker task? A closer parallel to disengaging from the non-target language would be disengaging from one nonverbal task in order to do a second task. This seems to map directly onto the prediction that bilinguals would have smaller switching costs in cued switching tasks. Indeed, Grundy et al. [3] point out that on switch trials participants must rapidly disengage from information that was relevant on the previous trial and refocus on information that was previously irrelevant. They go on to cite the bilingual advantages in switch costs reported by Prior and MacWhinney [28] and Prior and Gollan [29] as consistent with the hypothesis that bilinguals can disengage attention more rapidly than monolinguals. However, as shown in the meta-analysis by Paap [5] and in the meta-analysis of the shifting component by Lehtonen et al. [10] there is no compelling evidence that bilingual advantages in switching or mixing are different from zero. To rephrase, Grundy et al. [3] observed that *“constant monitoring and disengagement of attention from the non-target language are required to focus on the target language in the face of active competition”* p. 53. This seems to describe the executive control needed to reconfigure the intended task schema (e.g., from *speak Spanish* to *speak English*) and this may have an equivalent in nonverbal task shifting. Namely, having to monitor for and then encode the task cue and either switch task schema (e.g., *respond to* *color* to *respond to* *shape*) or stay on task (e.g., *continue to* *respond to* *color*). If a crucial aspect of both switching languages and switching nonverbal tasks is disengaging from the non-intended task, then bilingual advantages should occur in cued switching tasks. As noted above, such advantages rarely occur and meta-analytically the mean effect size is near zero.

Bilingual advantages in switch costs should not occur if language switching is controlled by a dedicated mechanisms rather than a domain-general mechanism. This is, of course, another debated topic, but intriguingly, a dissociation between switching languages and switching tasks within a single language was recently reported by Cattaneo, Costa, Gironell, and Calabria [30] in a study with Parkinson's disease patients. Patients and a matched control group completed both (1) a cued picture naming task that required random switches between Spanish and Catalan and (2) a task where the cue specified naming (always in the same language) the object or the action shown in a picture. In the first task, the between-language switching task, patients had increased switch costs and mixing costs compared to controls. In contrast, in the within-language switching task patients were impaired only in mixing costs. The key here is that switch costs are impaired only in the task that required switching languages. This pattern is consistent with the view that switch costs are driven by a mechanism specific to language switching and that this mechanism is localized in the basil ganglion (a region that is degraded by Parkinson's disease). If switching languages is governed by a control mechanism that is domain-specific, then it is not surprising that there was a non-significant correlation between the two types of switch costs (between-language versus within-language).

As Cattaneo et al. [30] observe, the literature of language switching has primarily focused on reactive control measured as switching costs, but control can also be exercised proactively as reflected in mixing costs. This, of course, neatly maps into Braver's DMC framework discussed earlier. If bilingual language control abilities are domain specific for reactive control (but domain-general for proactive control) then there is no reason to expect that practice at disengaging attention during language switching would transfer to a nonverbal interference task (e.g., a flanker task) and account for smaller SCEs. Such transfer would require that the same control mechanism was used in both tasks.

## 9. Linking SCEs in nonverbal interference tasks to language control

What is the implied relationship between SCEs in the flanker task and sequential effects in language production? Looking at trial-to-trial sequence effects in a flanker task would be like looking at word-to-word sequence effects in language production. One could speculate that during language production speakers must disengage attention from the word just uttered in order to plan and execute the next word. Indeed, Dell, Burger, and Svec [31] assert that the speaker must balance the various subtasks in utterance planning in order to *“activate the present, deactivate the past, and prepare to activate the future”* p. 123. But what is the argument that bilinguals receive more practice at this type of disengagement compared to monolinguals? Sevald and Dell [32] had participants produce sequences of four CVC words as many times as they could in eight seconds using the *parameter remapping paradigm* developed by Rosenbaum, Weber, Hazelett, and Hindorff [33]. Repetitions of the first consonant appear to cause greater competition (delays in responding and greater error rates) down stream, whereas repetitions of the final consonant produce facilitation. Thus, it was easier to produce a sequence including PICK TICK than one like PICK PIN. These sequential effects that produce benefits or costs depending on the location of the repetition within each word seem analogous to the benefits or costs observed in a flanker task contingent upon the congruency of the previous trial. To our knowledge there have not been any comparisons between bilinguals and monolinguals using the parameter remapping paradigm or other production tasks, but given that the stage of “deactivating the past” referred to by Dell et al. occurs continuously in monolingual language production there would be no reason for bilinguals to gain an edge in the speed of “disengaging attention”. Thus, if tested in a single-language context there is no obvious reason why bilinguals should have smaller sequential effects.

## 10. Other evidence that bilinguals are better at disengaging attention

Apart from SCEs, several other tasks have been cited as evidence that bilinguals can disengage attention more efficiently than monolinguals. These included cued switch costs, conjunctive visual search, ambiguous figures, and IOR. Some of these we critically reviewed in [6] and will be concisely referenced here. Friesen, Latman, Calvo, and Bialystok [34] concluded that the practice bilinguals receive at disengaging attention from the non-target language produces far transfer in the form of an enhanced ability to disengage attention from the distractors and more quickly find the target during conjunctive visual search. Ratiu, Hout, Walenchok, Azuma, and Goldinger [35] failed to replicate this result in three experiments as did Paap et al. [6] Furthermore, in a recently completed study we [36] also using a conjunctive visual search task (as similar to Friesen et al. [34] as we could instantiate based on the description in their methods section) we found no significant differences between bilinguals (n = 85) and monolinguals (n = 84). In fact, the language group means for both positive (target present) slope and negative (target absent) slope differed by less than 2 ms, t(167) = .40, *p* = .69 and t(167) = −.32, *p* = .75.

Chun-Fat-Yim, Sorge, and Bialystok [37] reported a bilingual advantage in an ambiguous figures task where participants were asked to guess, as soon as they could, the identity that the start object was morphing into. The authors suggested that bilinguals were better able to disengage attention from the salient features consistent with the start object in order to selectively attend to features consistent with the evolving object. In a close replication Paap et al. [6] found no significant differences between bilinguals (n = 79) and monolinguals (n = 44) and Bayes Factors provided substantial evidence for the null hypothesis.

Grundy et al. [3] also cite Mishra, Hilchey, Singh, and Klein's [38] report that high-proficiency bilinguals display IOR effects at earlier SOAs than low-proficiency bilinguals as support for the disengagement hypothesis. However, the Mishra et al. [38] study did not include monolinguals. In a study [39] that actually did compare bilinguals (n = 24) to monolinguals (n = 28) there were no group differences in the time course of IOR. Furthermore, in a replication and extension of their earlier work Saint-Aubin et al. (2018) [40] tested a large sample of English– French bilinguals and reported no effects of L2 proficiency on the IOR. They concluded that there is no reliable evidence that mastering a second language leads to faster or more potent disengagement of endogenous attention.

It is not our intention to discount all evidence that bilinguals are better at disengaging attention, merely to point out that the positive evidence highlighted by Grundy and Bialystok is difficult to replicate and, hence, not very compelling. Another intriguing outcome [41] said to be consistent with the disengagement hypothesis used a combined word identification/negative priming paradigm. In the first task of a couplet participants heard a word (e.g. “plum”) and saw four pictures presented in the four corners of an imaginary square. Their task was to identify the location of the target by pressing a corresponding button. On some trials one of the distractor pictures was phonologically similar to the target (e.g., “plus”) and acted as a competitor compared to unrelated control trials. The competitor is apparently activated by the shared phonology and is often the target of an anticipatory eye movement Fairly small samples (n = 30) of bilinguals and monolinguals showed equivalent degrees of competitor interference that were indexed as a slowing of the correct response on competitor trials versus control trials. This word identification task was immediately followed by a visual cue in one of the locations previously occupied by the pictures. Participants had to make a second response that indicated the location of the probe. This becomes a negative priming trial if the probe is presented in the location previously occupied by the competitor. Probe RT should be slower to the degree that the competing picture was inhibited during the word identification task. Only the monolingual group showed negative priming. Although two interpretations were considered Blumenfeld and Marian preferred the explanation that the residual inhibition may already have dissipated in the bilinguals, suggesting that bilinguals may return to a baseline activation state faster after inhibiting irrelevant information. If the rate at which inhibition decays (disengagement of inhibition?) is equated with the disengagement of attention control, then these results are relevant to our discussion.

In his review of this article Grundy noted that although no behavioral effects were observed in their Study 3 (long RSI), brain differences were observed. In several of our publications [42]–[45] we have discussed in detail and provided examples of the problems that accrue when neural differences between groups do not align with the behavioral differences. These problems occur because neural differences often suffer from valence ambiguity, that is, disagreements whether “more” implies better or worse functioning and also from “kind” ambiguity, that is, disagreements regarding what type of mental events the pattern of activation in a region-of-interest actually reflects. In our view neuroscience data clearly establishes that bilingual and monolingual brains are different, but cannot adjudicate interpretations of valence or kind.

## 11. Conclusion

The purpose of this commentary was to consider Grundy et al.'s hypothesis that ubiquitous bilingual language control leads to enhanced ability to disengage attention. Concerns about both empirical evidence and the interpretation of that evidence were raised. With regard to the specific hypothesis that bilinguals have smaller SCEs in nonverbal interference tasks like the flanker, we reviewed the results of 12 experiments from four different laboratories that showed robust CSEs in both the flanker and Simon task, but no significant language group differences. Thus, the smaller CSEs reported by Grundy et al. have yet to be replicated. However, Grundy et al. have asserted that Goldsmith and Morton's [4] null results (and presumably the others as well) are due to long RSIs that enable all participants to completely disengage attention from the previous trial. This argument was refuted by pointing out that all studies show robust SCEs which would not be expected if all, or even most, participants had sufficient time to fully disengage from the previous trial. Furthermore, we reviewed a study [22] on the time course of SCEs showing that these effects fade in the range of two to six seconds; a much longer RSI than the differences between the reviewed studies. In summary, the null results can reasonably be taken as failures to replicate and, at best, the research literature weakly supports the hypothesis that bilinguals have smaller SCEs.

Setting aside the weakness of the empirical evidence, it is of general interest to consider if the magnitude of SCEs in the standard flanker and Simon tasks reflect anything about the degree to which individuals or groups can adaptively improve performance in terms of global RT or accuracy. One important observation is that when congruent and incongruent trials are equiprobable (as is true in all of the experiments comparing bilinguals to monolinguals) the reduction in the interference effect when the previous trial was incongruent is caused by mirror-image costs and benefits such that global RT remains constant regardless of whether the SCE is large, small, or even zero. Another important result [13] is that although SCEs tend to be present in early blocks and then disappear. In contrast, effects of the proportion of congruent trials are sustained to the end of the session. This pattern fits Braver's DMC framework and shows that, at least for the Mayr and Awh data, that proactive effects adaptively improve performance and are maintained whereas reactive trial-to-trial effects in the form of SCEs disappear relatively quickly. Furthermore, the Cattaneo et al. [30] study of Parkinson's patients suggests that the control involved in reactive language switching languages is domain specific and not domain general. This too weakens the plausibility that practice in language switching transfers to a more general skill that enables more rapid disengagement of attention in other domains.

Setting aside both the weak empirical support for language group differences in the magnitude of SCEs and the conceptual problems just discussed in interpreting SCEs, other lines of evidence favoring the hypothesis that bilinguals are better at attention disengagement are less than compelling. These included cued switching tasks, slopes in conjunctive visual search tasks, ambiguous figures tasks, and inhibition-of-return. It may be more profitable for future research to focus on possible relationships between bilingual language control and proactive control.

## Footnotes

1 De Pisapia and Braver [24] describe an interesting neural net simulation of the DMC framework that assumes separate components of an ACC like structure that monitors for conflict over either a short time scale (to simulate reactive control) or over a long time scale (to simulate proactive control). The model successfully simulates the proportion congruent effect and the architectural step of having conflict monitored by independent units at two time scales enables the possibility of substantial proactive control in the absence of reactive control.
